# miR-429 promotes the proliferation of non-small cell lung cancer cells via targeting DLC-1

**DOI:** 10.3892/ol.2021.12806

**Published:** 2021-05-24

**Authors:** Peng Xiao, Wenliang Liu, Hui Zhou

Oncol Lett 12: 2163-2168, 2016; DOI: 10.3892/ol.2016.4904

Following the publication of this paper, it was drawn to the Editors’ attention by a concerned reader that certain of the western blotting data in the article (featured in Figs. 3B and 4A) were strikingly similar to data appearing in different form in a pair of other articles by different authors (which were already under consideration for publication or had already been published elsewhere at the time of the present article's submission.

Owing to the fact that the contentious data in the above article had already appeared in different form in other articles prior to its submission to Oncology Letters, the Editor has decided that this paper should be retracted from the Journal. The authors agreed with the retraction of the paper. The Editor apologizes to the readership for any inconvenience caused.

